# Creating a pediatric advisory board for engaging youth in pediatric health research: A case study

**DOI:** 10.1017/cts.2021.399

**Published:** 2021-03-30

**Authors:** Minerva Orellana, Miguel Valdez-Soto, Tabetha A. Brockman, Joyce E. Balls-Berry, Maria Guadalupe Zavala Rocha, Megan A. Allyse, Karen N. DSouza, Kirsten A. Riggan, Young Juhn, Christi Patten

**Affiliations:** 1 Mayo Clinic Graduate School of Biomedical Science, Rochester, MN, USA; 2 Center for Clinical and Translational Science, Community Engagement Program, Mayo Clinic, Rochester, MN, USA; 3 Biomedical Ethics Research Program, Mayo Clinic, Rochester, MN, USA; 4 Department of Obstetrics & Gynecology, Mayo Clinic, Rochester, MN, USA; 5 Division of Community Pediatric and Adolescent Medicine, Department of Pediatric and Adolescent Medicine, Mayo Clinic, Rochester, MN, USA; 6 Department of Psychiatry and Psychology, Mayo Clinic, Rochester, MN, USA

**Keywords:** Community-engaged research, youth, pediatric advisory board, human subjects research, community

## Abstract

Youth are an understudied population requiring additional safeguards when participating in research. Their input is necessary to facilitate participation and interest in studies. To address this, Mayo Clinic established one of the first pediatric advisory boards (PAB) comprised of 18 diverse youth aged 11–17. The PAB members participated in quarterly meetings (in person and then by video conference with the advent of COVID-19) where they provided feedback to researchers on recruitment strategies, study materials, and procedures. The PAB meetings fostered bidirectional conversations with researchers on several health research topics, including mental health. Youth advisory boards can promote engagement in pediatric research.

## Introduction

Youth are considered as a vulnerable population that requires additional protections to participate in human subjects research studies, such as providing assent and parent/guardian’s consent [1]. Many studies document recruitment strategies on how to engage youth in research studies in the study planning process [2–4]. However, many pediatric studies have low enrollment in clinical trials [5]. Greater youth participation in research study design, implementation, and interpretation might improve the quality and success of pediatric research by increasing engagement with the research team, providing age-appropriate study materials, and assessing comprehension of participation and risk/benefits [6]. In response, our NIH-funded Center for Clinical and Translational Science (CCaTS) developed one of the first pediatric research advisory boards (PAB) to overcome the knowledge gap in how to engage youth as meaningful partners in pediatric research.

A community-engaged research framework facilitates collaborative partnerships between academic researchers, community members, and patients who have shared interests or are connected geographically and have the same overall goal of increasing wellness [7]. A common platform for community engagement is the use of community advisory boards (CAB). CABs are typically comprised of adult patients and/or community members who advise investigators on research projects, including review of study materials and feedback on special considerations or risks/benefits to participants. CABs foster bidirectional conversations between investigators and community members [8]. Existing Mayo Clinic CABs have demonstrated success in addressing the health needs of the communities across the enterprise (Arizona, Florida, and Minnesota) [9]. However, to our knowledge, the creation of CABs that feature adolescent voices for pediatric research has not been reported in Clinical and Translational Science.

Our PAB was created with four primary goals: (1) designing pediatric research that represents the major health concerns of youth, (2) respecting the ability of youth to assent for research, (3) providing health benefits for youth through research, and (4) providing feedback to Mayo Clinic investigators on pediatric studies. This report describes the process of establishing the PAB at Mayo Clinic.

## Methods

### Setting

Rochester, MN USA.

### PAB Member Recruitment

Based on our experience with adult CABs, we aimed for 20 youth to join the PAB. The PAB coordinator was in charge of recruiting youth as members, with the goal of achieving a well-balanced diversity of age, gender, and ethnicity. Potential youth members were identified through existing relationships and referrals, as well as recommendations from established community partners with the Community Engagement Program team, including our adult CAB. Locations of recruitment included African-American churches, a youth-sponsored bike club, and a homeschooling network. Given the comparatively large immigrant population in our catchment area, we also engaged community liaisons to help communicate with parents/guardians of these potential PAB members. The project was explained in ongoing conversations with the youth and their parents/guardians. The PAB coordinator reached out to youth recommended by other stakeholders to gauge their interest before official invitations were distributed.

Interested adolescents were given personalized invitations to be part of the Mayo Clinic PAB. Once a parent/guardian’s consent was obtained, invitations were either mailed or hand-delivered in December 2018. Youth provided assent to participate in the PAB. Parents/guardians were asked to submit authorization for use of the youth’s image and video and co-sign a W-9 form for remuneration. The PAB members were paid $25 for each session they attended and a meal was provided.

### Training

Prior to giving feedback to researchers, PAB members were trained in the research process, human subject research, research ethics, and vulnerable populations. The first meeting in April 2019 was an introduction to the PAB members and staff. It also involved a presentation about the research process and special populations. The second meeting held in May 2019 focused on human research training and a speed information session with scientists. The July 2019 meeting involved learning about research ethics, vulnerable populations, and a researcher presentation. After human subjects research training in the initial sessions, the researchers presented their material of interest on July 2019, October 2019, and January 2020.

### Meeting Structure

In contrast to the traditional CAB structure, the team decided that the PAB would not have a Chair because of uncertainty that youth would be comfortable volunteering for this role. Instead, the PAB coordinator facilitated the meetings. Subsequently, there has been discussion of instituting a youth co-Chair for members who feel more confident or comfortable.

The PAB was established with quarterly meetings with the potential for expanding the frequency of meetings depending on the need of PAB members and researchers. Parents were not present at PAB meetings. All of the PAB meetings had a set agenda. The meetings were initiated with welcome and roundtable updates by the PAB coordinator with the PAB members. The speaker was then introduced, proceeded with their research presentation, and had an open discussion with the PAB members. The speakers were invited to come earlier to socialize with the PAB members if they were interested. Detailed minutes were taken at each meeting.

### Researcher Presentations

The availability of the PAB to researchers was promoted by the coordinator and other Community Engagement Program staff through word-of-mouth referrals and consultations. Subsequently, the PAB has been advertised through Mayo Clinic organizational websites, newsletters, and presentations.

The PAB coordinator first met with the researchers to see if their research and needs met the scope of the PAB. An invitation to speak at one of the PAB sessions was then extended. A presenter form was emailed to the researcher asking them to limit their presentation to five slides and three questions, given the age demographic of the PAB. If the PAB coordinator felt the questions were too difficult or would not be readily understood by the PAB members, the researcher was asked to reword and resubmit for review.

## Results

### Demographics

A total of 19 invitations to be part of the PAB were sent. All were accepted except one due to a work conflict. Our recruitment goal was reached with 9 girls and 9 boys aged 11–17 joining the PAB. All lived in Olmsted County or surrounding communities. Demographic information was missing for three members including one who moved out of the catchment area (Table 1). Quarterly PAB attendance ranged from 13 to 18 members with the exception of the January 2020 session which had 5 members.

**Table 1.** Demographics of pediatric advisory board

	Mean	SD
*Age (years)*	14.1	1.7
	**n**	**%**
*Race/Ethnicity*		
Black/African Ancestry/African-American	3	20%
White/Caucasian	4	27%
Hispanic/Latinx	3	20%
More than one (+2)	5	33%
*Education Level*		
Elementary	2	13%
Middle school	3	20%
High school	10	67%

### Feedback

The topics of each of the first five meetings of the PAB are presented in Table 2. Two of the three investigators were initially presented to the PAB specialized in pediatrics and the other in adolescent psychology. The PAB provided feedback to speakers such as comments to help increase awareness of research studies for adolescents. Some examples included how to direct research flyers or letters to the adolescent’s interest and not their parents. PAB members provided suggestions of locations to advertise research studies and to conduct focus groups. Many of the sessions were bidirectional with members advocating for health concerns in their age group, including mental health and vaping. Other examples are listed in Table 2.

**Table 2.** Meeting purpose and topic(s)

Date	Purpose	Topic(s)	Questions to the Pediatric Advisory Board (PAB)
April 2019	Welcome/Introduction	Introductory meeting for members to meet each other and the scientific team	Training sessions
May 2019	Human Subject Training	Training provided to PAB members on responsible conduct of research	Training sessions
July 2019	Learning about Vulnerable Populations and Research Ethics	Active discussion on traditionally vulnerable populations that have experienced historical mistrust related to health research	Training sessions
	Depression in Adolescents	Examples of creating awareness of the study in adolescents	What is the best way to create awareness of the study with teenagers?
October 2019	Experiences of Interpersonal Discrimination in Pediatric Settings	How to advertise the study letter to parents and adolescents Where to hold the focus groups Thoughts on safe and/or support space	What do you think about this letter that will be mailed to parents and adolescents? Any other guidelines do you all suggest we follow to keep this focus group a safe and supportive space?
January 2020	Thoughts on PAB Members Being in a Research Study	Thoughts on providing advice How interviews should be done Who would benefit from the study	How you feel about providing advice to researchers?
	Study on Attitudes, Beliefs, Behaviors, and Clinical Practices Related to Youth Marijuana and Cannabidiol use	A series of questions pertaining to the comfort levels of someone they know at a doctor’s office with their parent discussing marijuana use	How do you think a parent would feel about marijuana use? What is it about the parent being there would take away from that open communication between the child and doctor?

## Discussion

Children and adolescents are defined as a vulnerable research population who require special considerations. However, our case study indicates that researchers can seek out youth for their input to better improve the recruitment and retention of children and adolescents in their studies.

Other study-specific youth advisory boards focused on limited health topics or improving organizations [10–13]. Some youth advisory boards are comprised of adult members discussing topics pertaining to youths [14]. A few advisory boards are based in hospitals to help improve pediatric treatments including at Children’s Minnesota, Boston Children’s Hospital, and Children’s Mercy in Kansas City [15–17]. To our knowledge, the Mayo Clinic PAB in Rochester, Minnesota is one of the first of its kind. This is the first mention of a PAB created for bidirectional engagement, as well as advising on research studies focusing on youth participants in Clinical and Translational Science.

The PAB was created to be diverse and reflect the demographics of the US population and not only of Rochester, Minnesota. It is intended for researchers to seek out feedback for improving recruitment or reviewing study material for studies focused on adolescents. Researchers left the meetings with suggestions that would be implemented in their studies.

The PAB has been featured in both youth and adult-focused public media outlets for its role in creating opportunities for youth to have a voice in health-related research [18–20]. Additionally, the PAB provided adolescents a voice on studies featuring people like them and for discussion of health disparities prevalent in their own communities. Other institutions interested in conducting community-engaged research, particularly pediatric research, may also benefit from engagement with PAB.

### Limitations

Given the lack of precedence for the PAB model, there were initial challenges, including education of the PAB members to establish a base of knowledge from which to provide feedback. To understand members’ experience, a study was conducted to assess initial challenges and build upon the successes of the PAB. Despite achieving gender and racial/ethnic diversity of members, there is a need for more Asian representation on the board. We aim to include youth who do not identify within traditional gender roles. In time, we hope to address this as future spots are made available on the PAB.

### Future Directions

The PAB continues to meet quarterly and has transitioned to a video conferencing format following COVID-19 guidelines. Currently, there is a waiting list for membership. Future directions include transitioning of graduating PAB members into the Mayo Clinic CAB or future inclusion as an observer. Feedback can include helping researchers adapt to continue researching during these unprecedented times. PAB members could take more active roles in the entire research process such as initiating their own research projects on their topics of interest.
